# Overlap Syndrome of Primary Sjögren Syndrome with Antineutrophil Cytoplasmic Antibody (ANCA)-Associated Vasculitis Based on the American College of Rheumatology (ACR)/European Alliance of Associations for Rheumatology (EULAR) Criteria

**DOI:** 10.3390/diagnostics15091099

**Published:** 2025-04-25

**Authors:** Hyun Joon Choi, Jang Woo Ha, Jason Jungsik Song, Yong-Beom Park, Sang-Won Lee

**Affiliations:** 1 Department of Medicine, Yonsei University College of Medicine, Seoul 03722, Republic of Korea; 2 Division of Rheumatology, Department of Internal Medicine, Yongin Severance Hospital, Yonsei University College of Medicine, Yongin 16995, Republic of Korea; 3 Division of Rheumatology, Department of Internal Medicine, Yonsei University College of Medicine, Seoul 03722, Republic of Korea; 4 Institute for Immunology and Immunological Diseases, Yonsei University College of Medicine, Seoul 03722, Republic of Korea

**Keywords:** overlap syndrome, Sjögren syndrome, antineutrophil cytoplasmic antibody, vasculitis, reclassification

## Abstract

**Background/Objectives**: The overlap syndrome of primary Sjögren syndrome (pSS) with antineutrophil cytoplasmic antibody (ANCA)-associated vasculitis (AAV) (OvSD/pSS/AAV) has been reported in other studies. This study applied the new criteria for AAV proposed by the American College of Rheumatology/European Alliance of Associations for Rheumatology in 2022 (the ACR/EULAR criteria) to patients with pSS presenting signs and symptoms suggestive of small- and medium-vessel vasculitis. It also investigated the overall frequency of OvSD/pSS/AAV and the major contributing factors to its reclassification. **Methods**: This study included 116 patients with pSS from March 2005 to December 2020, according to the inclusion criteria, and defined signs and symptoms suggestive of small- or medium-vessel vasculitides as lung parenchymal lesions supporting AAV, peripheral neuropathy, and suspected renal vasculitis. The classification could be made when the total scores for microscopic polyangiitis (MPA) and granulomatosis with polyangiitis (GPA) are ≥5 points and the eosinophilic GPA (EGPA) score is ≥6 points. **Results**: The median age of the patients was 56.0 years, and 101 patients (87.1%) were women. In total, 95, 12, and 37 patients had lung parenchymal lesions supporting AAV, peripheral neuropathy, and suspected renal vasculitis, respectively. According to the ACR/EULAR criteria for AAV, 35 of 116 (30.2%) patients were reclassified as having OvSD/pSS/AAV. Among these 35 patients, 4 were reclassified as having both OvSD/pSS/MPA and OvSD/pSS/GPA and 1 as having both OvSD/pSS/MPA and OvSD/pSS/EGPA simultaneously. The major contributing factor to the reclassification of OvSD/pSS/AAV was ANCA positivity. **Conclusions**: The overall frequency of the reclassification of OvSD/pSS/AAV was 30.2% in pSS patients presenting signs and symptoms suggestive of small- and medium-vessel vasculitis. Its likelihood increased according to ANCA positivity.

## 1. Introduction

Primary Sjögren syndrome (pSS) is a progressive autoimmune disease, characterized by exocrinopathy, particularly, the involvement of salivary and lacrimal glands, resulting in dryness in the eyes and mouth [1]. Furthermore, approximately 75% of patients with pSS may exhibit extraglandular manifestations: nonspecific symptoms including arthralgia, arthritis, Raynaud’s phenomenon, and fatigue and systemic complications including interstitial lung disease (ILD), tubulointerstitial nephritis (TIN) or glomerulonephritis (GN), peripheral neuropathies, and lymphomas [2,3,4,5].

Additionally, systemic vasculitis is one of the most frequent extraglandular manifestations in patients with pSS, and further, small vessel vasculitis, particularly cryoglobulinemic vasculitis is known as the most common vasculitis in patients with pSS [6,7]. On the other hand, the clinical features of overlap syndrome (OvSD) of pSS with antineutrophil cytoplasmic antibody (ANCA)-associated vasculitis (AAV) (OvSD/pSS/AAV), which is an infrequent type of pSS-related vasculitis, have been reported as follows: (i) a detection rate of ANCA positivity at 10% to 20% immunologically; (ii) a low proportion of patients reclassified as OvSD/pSS/AAV; (iii) the strong association between myeloperoxidase (MPO)-ANCA and reclassification as OvSD/pSS/AAV; (iv) the relatively high rate of kidney involvement; (v) the concomitant occurrence of pSS with AAV [8,9,10]. However, in real clinical practice, OvSD/pSS/AAV has a low index of suspicion among patients with pSS regardless of ANCA positivity. This is because, when patients with pSS have ILD, TIN, GN, or peripheral neuropathy, these are considered systemic complications of pSS rather than symptoms of pSS-related AAV.

In 2022, the American College of Rheumatology (ACR) and the European Alliance of Associations for Rheumatology (EULAR) proposed new classification criteria (the ACR/EULAR criteria) for microscopic polyangiitis (MPA), granulomatosis with polyangiitis (GPA), and eosinophilic GPA (EGPA) [11,12,13,14]. Given clinical situations that limit the classification of OvSD/pSS/AAV, this study applied the ACR/EULAR criteria for AAV to patients who were diagnosed with pSS according to the 2016 ACR/EULAR criteria for pSS and had signs and symptoms suggestive of small- and medium-vessel vasculitis. Additionally, this study investigated the overall frequency of OvSD/pSS/AAV and the major contributing factors to its reclassification.

## 2. Materials and Methods

### 2.1. Patients

The present study screened 680 patients with pSS retrospectively according to the following inclusion criteria: (i) patients diagnosed with pSS at the Division of Rheumatology, Department of Internal Medicine, Yonsei University College of Medicine, Severance Hospital, from March 2005 to December 2020; (ii) patients fulfilling the 2016 ACR/EULAR classification criteria for pSS [15]; (iii) patients with well-written medical records wherein clinical data at pSS diagnosis and during follow-up could be retrospectively and easily obtained; (iv) patients with the results of tests for ANCA at pSS diagnosis; (v) patients without concomitant malignancies or serious infectious diseases [16,17]; (vi) patients without concomitant autoimmune diseases affecting ANCA false positivity, for instance, Crohn’s disease, ulcerative colitis, or primary sclerosing cholangitis [18]; (vii) patients without exposure to drugs affecting ANCA false positivity such as propylthiouracil and hydralazine [19,20]; (viii) patients presenting signs and symptoms suggestive of small- or medium-vessel vasculitides including lung parenchymal lesions supporting AAV, peripheral neuropathy, or suspected renal vasculitis. According to the 2016 ACR/EULAR classification criteria for pSS, anti-SSA/Ro is an autoantibody closely associated with the diagnosis of pSS. A positive Schirmer’s test is defined as ≤5 mm/5 min in at least one eye. A positive unstimulated whole salivary flow test is defined as a flow rate of ≤0.1 mL per minute. A positive ocular staining score is defined as a score of ≥5 in at least one eye, based on the assessment of corneal and conjunctival staining.

**Figure 1:**
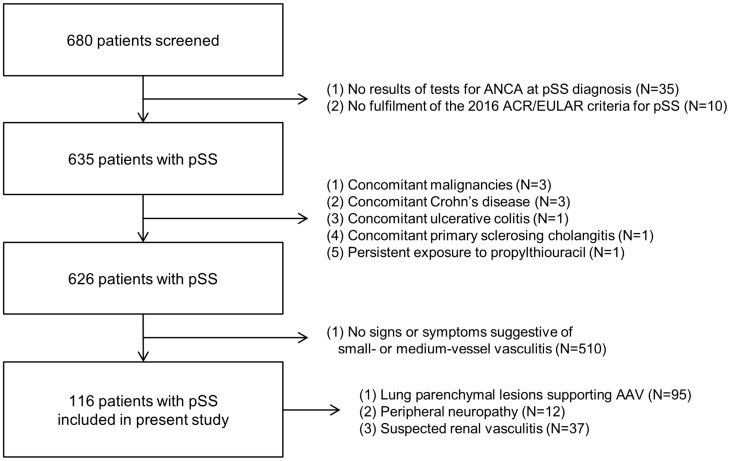
Algorithm of patients’ selection. ANCA: antineutrophil cytoplasmic antibody; pSS: primary Sjögren syndrome; ACR: American College of Rheumatology; EULAR: European Alliance of Associations for Rheumatology.

Among the 680 patients with pSS, 35 were excluded owing to absent results of tests for ANCA at pSS diagnosis, and 10 were excluded owing to no fulfilment of the 2016 ACR/EULAR criteria for pSS. The additional exclusions included three patients with concomitant malignancies, one with Crohn’s disease, one with ulcerative colitis, and one with persistent exposure to propylthiouracil. Subsequently, among the remaining 626 patients, 510 were excluded owing to absent signs of symptoms suggestive of small- or medium-vessel vasculitides. Finally, 116 patients with pSS were included in the present study owing to the following conditions: lung parenchymal lesions supporting AAV (*n* = 95), peripheral neuropathy (*n* = 12), and suspected renal vasculitis (*n* = 37) (Figure 1).

### 2.2. Ethical Approval

The present study was approved by the institutional review board (IRB) of Severance Hospital (Seoul, Republic of Korea; IRB No. 4-2023-1297). Given the retrospective design of this study and the use of anonymized patient data, the requirement for written informed consent was waived by the IRB.

### 2.3. Signs and Symptoms Suggestive of Small- or Medium-Vessel Vasculitis

In this study, signs and symptoms suggestive of small- or medium-vessel vasculitides were defined as three clinical manifestations including lung parenchymal lesions supporting AAV, peripheral neuropathy, and suspected renal vasculitis. Lung parenchymal lesions supporting AAV included two findings: pulmonary fibrosis or ILD suggesting MPA and pulmonary nodules, mass, and cavitation indicating GPA [11,12]. Moreover, lung parenchymal lesions supporting AAV and peripheral neuropathy were confirmed by high-resolution computed tomography of the lungs and nerve conduction velocity studies, respectively [14]. Suspected renal vasculitis was defined as RBC cast-related hematuria, >10% dysmorphic RBC hematuria, or 2+ hematuria and 2+ proteinuria on a urine stick [21].

### 2.4. Clinical Data at Diagnosis

The numbers of patients having lung parenchymal lesions supporting AAV; pulmonary fibrosis or ILD suggesting MPA; pulmonary nodules, mass, and cavitation indicating GPA; peripheral neuropathy; and suspected renal vasculitis at pSS diagnosis were counted. Age and sex were collected as demographic data, and the items of the 2016 ACR/EULAR criteria for pSS were investigated [1,2,15]. According to the ACR/EULAR criteria for AAV, the results of tests for perinuclear (P)-ANCA, cytoplasmic (C)-ANCA, MPO-ANCA, and proteinase 3 (PR3)-ANCA were all accepted [11,12,13]. P-ANCA and C-ANCA were detected by an indirect immunofluorescence assay on ethanol-fixed neutrophils, whereas MPO-ANCA and PR3-ANCA were measured by using an antigen-specific immunoassay based on enzyme-linked immunosorbent assay techniques. The results of laboratory tests were also recorded (Table 1).

### 2.5. The ACR/EULAR Criteria for AAV

The ACR/EULAR criteria for MPA, GPA, and EGPA are composed of a total score that summates the points differently assigned to each item. The classification could be made when the total scores for MPA and GPA are 5 points or more and that for EGPA is 6 points or more. Additionally, two mandatory requirements must be met before applying these criteria: the presence of evidence of small- or medium-vessel vasculitis and the absence of other medical conditions mimicking AAV [11,12,13].

### 2.6. Statistical Analyses

All the statistical analyses were performed using SPSS Statistics for Windows, version 26 (IBM Corp., Armonk, NY, USA). Continuous and categorical variables were expressed as medians (Q1–Q3) and numbers (percentages), respectively. Significant differences in categorical variables were assessed using the chi-square test and Fisher’s exact test, while differences in medians of continuous variables with non-normal distributions were evaluated using the Mann–Whitney U test. *p*-values < 0.05 were considered statically significant.

## 3. Results

### 3.1. Characteristics of Patients with pSS Presenting Signs and Symptoms Suggestive of Small- or Medium-Vessel Vasculitis at pSS Diagnosis

Among the 116 pSS patients presenting signs and symptoms suggestive of small- and medium-vessel vasculitis, 95, 12, and 37 had lung parenchymal lesions supporting AAV, peripheral neuropathy, and suspected renal vasculitis, respectively. Moreover, 89 and 65 exhibited pulmonary fibrosis or ILD suggesting MPA and pulmonary nodules, mass, and cavitation indicating GPA on chest imaging, and further, 59 showed both of them. The median age of the patients with pSS was 56.0 years and 87.1% were females. Regarding the items of the 2016 ACR/EULAR criteria for pSS, anti-SSA/Ro, was detected in 111 (95.7%) patients, and typical minor salivary gland histological findings were found in 5 (4.3%) patients who did not have anti-SSA/Ro. Among the 116 patients, 72 (62.1%) and 83 (71.6%) exhibited positive results of Schirmer’s test and unstimulated whole saliva flow rate, respectively. Additionally, ANCA was detected as positive in 30 (25.9%) patients: 28 (24.1%) had MPO-ANCA (or P-ANCA), and 4 (3.4%) had PR3-ANCA (or C-ANCA). The median ESR and CRP levels were 47.0 mm/h and 1.9 mg/L, respectively. The remaining laboratory results are described in Table 1.

### 3.2. Application of the ACR/EULAR Criteria for MPA to Patients with pSS Presenting Signs and Symptoms Suggestive of Small- and Medium-Vessel Vasculitis

Regarding the clinical criteria, seven (6.0%) patients exhibited symptoms of nasal involvement. Regarding the laboratory, imaging, and biopsy criteria, the most often met item was fibrosis or ILD on chest imaging (76.7%), followed by MPO-ANCA (or P-ANCA) positivity (24.1%). Pauci-immune glomerulonephritis was confirmed on biopsy in one patient. Additionally, four (3.4%) and eight (6.9%) had PR3-ANCA (or C-ANCA) and serum eosinophil count ≥ 1000/μL, respectively. Among the 116 patients with pSS, 27 (23.3%) achieved total scores ≥ 5 and could be reclassified as having OS of pSS with MPA (OvSD/pSS/MPA) [11] (Table 2).

### 3.3. Application of the ACR/EULAR Criteria for GPA to Patients with pSS Presenting Signs and Symptoms Suggestive of Small- and Medium-Vessel Vasculitis

Regarding the clinical criteria, 7 (6.0%) and 22 (19.0%) patients exhibited symptoms of nasal involvement and cartilaginous involvement, respectively. In addition, three (2.6%) patients met the item of conductive or sensorineural hearing loss. Regarding the laboratory, imaging, and biopsy criteria, the most frequently observed plus-scored item was pulmonary nodules, mass, or cavitation (56.0%), followed by nasal/paranasal sinusitis or mastoiditis on imaging (11.2%) and granuloma, granulomatous inflammation, or giant cells on biopsy (4.3%). PR3-ANCA (or C-ANCA) was detected in four (3.4%) patients. Conversely, 28 (24.1%) and 8 (6.9%) patients received −1 and −4 scores owing to MPO-ANCA (or P-ANCA) positivity and serum eosinophil count ≥ 1000/μL, respectively. Among the 116 patients with pSS, 11 (9.5%) achieved total scores ≥ 5 and could be reclassified as having OS of pSS with GPA (OvSD/pSS/GPA) [12] (Table 3).

### 3.4. Application of the ACR/EULAR Criteria for EGPA to Patients with pSS Presenting Signs and Symptoms Suggestive of Small- and Medium-Vessel Vasculitis

Regarding the clinical criteria, 15 (12.9%), 1 (0.9%), and 5 (4.3%) patients met the items of obstructive airway disease, nasal polyps, and mononeuritis multiplex, respectively. Regarding the laboratory, imaging, and biopsy criteria, eight (6.9%) patients received a +5 score owing to serum eosinophil count ≥ 1000/μL. Conversely, 4 (3.4%) and 78 (67.2%) received −3 and −1 scores due to PR3-ANCA (or C-ANCA) positivity and hematuria, respectively. Among the 116 patients with pSS, 2 (1.7%) achieved total scores ≥ 6 and could be reclassified as having OS of pSS with EGPA (OvSD/pSS/EGPA) [13] (Table 4).

### 3.5. Frequency of OvSD/pSS/AAV Among Patients with pSS Presenting Signs and Symptoms Suggestive of Small- and Medium-Vessel Vasculitis

Among the 116 pSS patients presenting signs and symptoms suggestive of small- and medium-vessel vasculitis, 27 (23.3%), 11 (9.5%), and 2 (1.7%) patients were reclassified as having OvSD/pSS/MPA, OvSD/pSS/GPA, and OvSD/pSS/EGPA, respectively.

Of these patients reclassified as having OvSD/pSS/AAV, four were reclassified as having OvSD/pSS/MPA and OvSD/pSS/GPA simultaneously, whereas one was reclassified as having OvSD/pSS/MPA and OvSD/pSS/EGPA simultaneously (Tables S1 and S2). As such, 35 patients were reclassified as having OvSD/pSS/AAV; thus, the overall frequency of the reclassification of OvSD/pSS/AAV was 30.2%.

### 3.6. Comparison of Variables Between Patients with OvSD/pSS/AAV and Those Without

When pSS patients presenting signs and symptoms suggestive of small- and medium-vessel vasculitis were divided into two groups according to the diagnosis of OvSD/pSS/AAV, 35 patients belonged to the group OvSD/pSS/AAV. Comparing the variables between the two groups, patients with OvSD/pSS/AAV were notably more likely to be ANCA positive (82.9% vs. 1.2%, *p* < 0.001), MPO-ANCA (or P-ANCA) positive (77.1% vs. 1.2%, *p* < 0.001), and PR3-ANCA (or C-ANCA) positive (11.4% vs. 0%, *p* = 0.002) than those without. However, no significant differences in lung parenchymal lesions supporting AAV, peripheral neuropathy, and suspected renal vasculitis between the two groups were observed (Table 5).

## 4. Discussion

In the present study, we applied the ACR/EULAR criteria for AAV to patients who were diagnosed with pSS according to the 2016 ACR/EULAR criteria for pSS and had signs and symptoms suggestive of small- and medium-vessel vasculitis.

This study describes in more detail the five pSS patients reclassified as having two forms of OvSD/pSS/AAV simultaneously. Of the 35 patients reclassified as having OvSD/pSS/AAV, 4 were reclassified as having OvSD/pSS/MPA and OvSD/pSS/GPA simultaneously. In terms of the criteria for MPA, the items of MPO-ANCA (or P-ANCA) positivity and MPA-related lung lesions mainly contributed to the reclassification as having OvSD/pSS/MPA. Whereas, in terms of the criteria for GPA, the items of cartilaginous involvement, GPA-related lung lesions, and PR3-ANCA (or C-ANCA) positivity primarily contributed to the reclassification as having OvSD/pSS/GPA (Table S1) [11,12,13]. Additionally, one patient was reclassified as having OvSD/pSS/MPA and OvSD/pSS/EGPA simultaneously. In terms of the criteria for MPA, the patient achieved a total score of 5 owing to the items of MPO-ANCA (or P-ANCA) positivity (+6), MPA-related lung lesions (+3), and serum eosinophil count ≥ 1000/μL (−4) and could be reclassified as having OvSD/pSS/MPA. In terms of the criteria for EGPA, the patient achieved a total score of 7 owing to the items of obstructive airway disease (+3), serum eosinophil count ≥ 1000/μL (+5), and PR3-ANCA (or C-ANCA) positivity (−1) and could be reclassified as having OvSD/pSS/EGPA (Table S2) [11,12,13]. Given that the treatment strategy for MPA and GPA is the same, dealing with patients reclassified as having both OvSD/pSS/MPA and OvSD/pSS/GPA simultaneously might not be difficult. However, because the treatment strategies of MPA and EGPA are quite different, some confusion might be expected in coping with AAV during exacerbation in patients reclassified as having both OvSD/pSS/MPA and OvSD/pSS/EGPA [22,23,24].

In this study, both radiological findings including pulmonary fibrosis or ILD suggesting MPA and pulmonary nodules, mass, and cavitation indicating GPA critically contributed to the reclassification of OvSD/pSS/MPA and OvSD/pSS/GPA as described in Table 2 and Table 3 and Tables S1 and S2. To reaffirm the predictive potential of lung parenchymal lesions supporting AAV for the reclassification of OvSD/pSS/AAV in pSS patients, five patients without ANCA who were reclassified as having OvSD/pSS/GPA were selected, and the items contributing to the reclassification were investigated. All the five patients had pulmonary nodules, mass, and cavitation on chest imaging, and four and three exhibited nasal involvement (congestion) and paranasal sinusitis, respectively. All the five patients achieved total scores of 5 or more and could be reclassified as having OvSD/pSS/GPA (Table S3) [12]. Therefore, lung parenchymal lesions supporting AAV, particularly, pulmonary nodules, mass, and cavitation, could contribute to the reclassification of OvSD/pSS/GPA in pSS patients without ANCA.

Additionally, we introduced one patient who did not have ANCA but was reclassified as having OvSD/pSS/EGPA. The patient was included in this study owing to the presence of peripheral neuropathy; however, the typical finding of mononeuritis multiplex was not found in the nerve conduction velocity study. According to the ACR/EULAR criteria for EGPA, the patient achieved a total score of 8 owing to asthma (+3) and eosinophil count ≥ 1000/μL (+5) despite the absence of mononeuritis multiplex and could be reclassified as OvSD/pSS/EGPA (Table S4) [13]. As such, five patients and one patient without ANCA were newly reclassified as having OvSD/pSS/GPA and OvSD/pSS/EGPA, respectively.

Among the 116 patients included in this study, 35 patients were newly reclassified as having OvSD/pSS/AAV. Given that ANCA was detected in 30 patients and a total of 6 patients without ANCA were newly reclassified as having OvSD/pSS/AAV, 1 patient with ANCA positivity failed to be reclassified as having OvSD/pSS/AAV. The patient had MPO-ANCA (or P-ANCA) and serum eosinophil count ≥ 1000/μL and exhibited pulmonary nodules, paranasal sinusitis, and hematuria at pSS diagnosis. The patient achieved total scores of 2, −2, and 4 according to the ACR/EULAR criteria for MPA, GPA, and EGPA, respectively, and finally could not be reclassified as having OvSD/pSS/AAV (Table S5) [11,12,13]. Therefore, we conclude that the role of the item of ANCA positivity in the reclassification of OvSD/pSS/AAV may be critical but not essential.

To date, the reclassification of OvSD/pSS/AAV has not been recognized as a significant issue in patients with pSS. The most important reason may be the official publication of the ACR/EULAR criteria for AAV. Unlike the 2012 revised Chapel Hill Consensus Conference for the Nomenclature of Vasculitides [25] or the 2007 European Medicine Agency algorithm for AAV [21], the features of the new criteria to infer the possibility of AAV in pSS patients may be hypothesized as follows: (i) the augmented role of MPO-ANCA (or P-ANCA) and PR3-ANCA (or C-ANCA) in the reclassification of MPA and GPA; (ii) the newly approved role of both P-ANCA and C-ANCA detected by an indirect immunofluorescence assay in the reclassification of MPA, GPA, and EGPA; (iii) the newly proposed lung lesions (fibrosis or ILD on chest imaging) in the reclassification of MPA; (iv) the emphasized role of serum eosinophilia in the reclassification of EGPA; and (v) the reduced role of histological findings [11,12,13,14].

Recognition of OvSD/pSS/AAV in patients with pSS is clinically essential. The most important reason is that the disease course and treatment strategies for pSS and AAV are quite different. First, in terms of all-cause mortality, the overall survival rate of Korean patients with pSS was reported as 99.0%, and the most common causes of death were respiratory diseases and malignancies [26]. However, the overall survival rate of Korean patients with AAV was reported to range from 11.9% to 13.2%, and the main cause of death was infectious diseases [27,28,29]. Second, in terms of systemic complications, both diseases are similar in that they can affect almost any organ. However, pSS has a relatively high frequency of hematological complications such as monoclonal gammopathy and B cell lymphoma or central nervous system involvement, whereas AAV has a relatively high frequency of kidney involvement and cardiovascular diseases [30,31]. Third, in terms of treatment, there are significant differences in treatment strategies according to the affected organs, definition of severity, and therapeutic drug regimens between the two diseases [22,23,24,32]. Therefore, we believe that the active discovery of OvSD/pSS/AAV patients among patients with pSS presenting signs and symptoms suggestive of small- and medium-vessel vasculitis may have the advantage of providing better treatment opportunities with two perspectives of both pSS and AAV to patients with new clinical manifestations after pSS diagnosis.

One advantage of this study is that it is the first to investigate the frequency of OvSD/pSS/AAV and the contributing factors of its reclassification by applying the newly proposed AAV criteria to patients with pSS presenting signs and symptoms suggestive of small- and medium-vessel vasculitis. Another advantage of this study is that it could provide opportunities to approach newly developed systemic complications with two perspectives of pSS and AAV by demonstrating the possibility of the reclassification of OvSD/pSS/AAV in patients already diagnosed with pSS.

However, this study has several limitations. This study was retrospectively conducted, and thus, the possibility of missing data could not be excluded. Additionally, the retrospective study design left three issues: first, hoarseness and stridor were recognized as cartilage involvement; second, nasal congestion was recognized as nasal passage involvement; and third, situations other than EGPA that could cause peripheral eosinophilia could not be ruled out. For these reasons, the overall frequency of OvSD/pSS/AAV might have been overestimated. In addition, due to the limitation of being a single-center study, the number of patients included in this study was not sufficient for generalizing the results of this study and applying them to patients with pSS immediately. On the other hand, although ILD contributed to the reclassification of OvSD/pSS/MPA, we could not unveil its clinical significance in the comparative analysis (Table 5), which might be due to the ignorance of the probability of ILD as the systemic complication of pSS rather than an MPA manifestation. However, it could be said that this limitation might enhance the accuracy of the diagnosis of both pSS and OvSD/pSS/AAV and minimize interobserver variation. We believe that a prospective and longitudinal future study with more pSS patients will provide more reliable information on the clinical implications of the reclassification of OvSD/pSS/AAV and increase the possibility of mining new biomarkers for predicting OvSD/pSS/AAV [33].

## 5. Conclusions

This study was the first to demonstrate that among patients with pSS presenting signs and symptoms suggestive of small- and medium-vessel vasculitis, the overall frequency of OvSD/pSS/AAV was 30.2% and that the major contributing factor to the reclassification of OvSD/pSS/AAV was any ANCA positivity. Furthermore, this study reminds us that discovering OvSD/pSS/AAV would provide better treatment opportunities with two perspectives of both pSS and AAV to patients with new clinical manifestations after pSS diagnosis.

## Figures and Tables

**Table 1:** Characteristics of patients with pSS presenting signs and symptoms suggestive of small- or medium-vessel vasculitis (*n* = 116).

Variables	Values
At pSS diagnosis	
Signs and symptoms suggestive of small- or medium-vessel vasculitis (*n*, (%))	
Lung parenchymal lesions supporting AAV	95 (81.9)
Pulmonary fibrosis or ILD on chest imaging	89 (76.7)
Pulmonary nodules, mass, and cavitation on chest imaging	65 (56.0)
Peripheral neuropathy	12 (10.4)
Suspected renal vasculitis	37 (31.9)
Demographic data	
Age (years)	56.0 (50.3–62.8)
Female sex (*n*, (%))	101 (87.1)
Items of the 2016 ACR/EULAR criteria for pSS (*n*, (%))	
Labial salivary gland with focal lymphocytic sialadenitis and focus score of ≥1 foci/4 mm^2^	5 (4.3)
Anti-SSA/Ro positive	111 (95.7)
Ocular staining score ≥ 5 in at least 1 eye	1 (0.9)
Schirmer’s test ≤ 5 mm/5 min in at least 1 eye	72 (62.1)
Unstimulated whole saliva flow rate < 0.1 mL/minute	83 (71.6)
Total scores ≥ 4	116 (100.0)
ANCA positive (*n*, (%))	
ANCA positive	30 (25.9)
MPO-ANCA (or P-ANCA) positive	28 (24.1)
PR3-ANCA (or C-ANCA) positive	4 (3.4)
Laboratory results	
White blood cell count (/mm^3^)	5920.0 (4705.0–7792.5)
Hemoglobin (g/dL)	12.7 (11.6–14.0)
Platelet count (×1000/mm^3^)	250.0 (190.0–312.5)
Blood urea nitrogen (mg/dL)	14.1 (10.3–17.8)
Serum creatinine (mg/dL)	0.7 (0.6–0.8)
Aspartate aminotransferase (IU/L)	24.5 (20.3–34.0)
Alanine aminotransferase (IU/L)	19.0 (14.0–32.0)
Total protein (g/dL)	7.4 (6.9–7.9)
Serum albumin (g/dL)	4.1 (3.7–4.4)
Acute phase reactants	
ESR (mm/h)	47.5 (26.3–75.0)
CRP (mg/L)	1.9 (0.7–5.4)
Complements	
C3 (mg/dL)	105.8 (95.2–118.3)
C4 (mg/dL)	24.0 (17.8–29.2)

Values are expressed as a median (Q1–Q3) or *n* (%). pSS: primary Sjögren syndrome; AAV: ANCA-associated vasculitis; ANCA: antineutrophil cytoplasmic antibody; ILD: interstitial lung disease; ACR: American College of Rheumatology; EULAR: European Alliance of Associations for Rheumatology; MPO: myeloperoxidase; P: perinuclear; PR3: proteinase 3; C: cytoplasmic; ESR: erythrocyte sedimentation rate; CRP: C-reactive protein; C3: complement 3; C4: complement 4.

**Table 2:** Application of the ACR/EULAR criteria for MPA to patients with pSS presenting signs and symptoms suggestive of small- and medium-vessel vasculitis (*n* = 116).

Variables		Values
At pSS diagnosis	**Score**	**(*n* (%))**
Items for the ACR/EULAR criteria for MPA		
Clinical criteria		
Nasal involvement (discharge, ulcers, crusting, congestion, septal defect/perforation)	−3	7 (6.0)
Laboratory, imaging, and biopsy criteria		
MPO-ANCA (or P-ANCA) positive	+6	28 (24.1)
Fibrosis or ILD on chest imaging	+3	89 (76.7)
Pauci-immune glomerulonephritis on biopsy	+3	1 (0.9)
PR3-ANCA (or C-ANCA) positive	−1	4 (3.4)
Serum eosinophil count ≥ 1000/μL	−4	8 (6.9)
Patients with total score ≥ 5 (*n* (%))		27 (23.3)

ACR: American College of Rheumatology; EULAR: European Alliance of Associations for Rheumatology; MPA: microscopic polyangiitis; pSS: primary Sjögren syndrome; MPO: myeloperoxidase; ANCA: antineutrophil cytoplasmic antibody; P: perinuclear; ILD: interstitial lung disease; PR3: proteinase 3; C: cytoplasmic.

**Table 3:** Application of the ACR/EULAR criteria for GPA to patients with pSS presenting signs and symptoms suggestive of small- and medium-vessel vasculitis (*n* = 116).

Variables		Values
At pSS diagnosis	**Score**	**(*n* (%))**
Items for the ACR/EULAR criteria for GPA		
Clinical criteria		
Nasal involvement (discharge, ulcers, crusting, congestion, septal defect/perforation)	+3	7 (6.0)
Cartilaginous involvement	+2	22 (19.0)
Conductive or sensorineural hearing loss	+1	3 (2.6)
Laboratory, imaging, and biopsy criteria		
PR3-ANCA (or C-ANCA) positivity	+5	4 (3.4)
Pulmonary nodules, mass, or cavitation	+2	65 (56.0)
Granuloma, granulomatous inflammation, or giant cells on biopsy	+2	5 (4.3)
Nasal/paranasal sinusitis or mastoiditis on imaging	+1	13 (11.2)
Pauci-immune glomerulonephritis on biopsy	+1	1 (0.9)
MPO-ANCA (or P-ANCA) positivity	−1	28 (24.1)
Serum eosinophil count ≥ 1000/μL	−4	8 (6.9)
Patients with total score ≥ 5 (*n* (%))		11 (9.5)

ACR: American College of Rheumatology; EULAR: European Alliance of Associations for Rheumatology; GPA: granulomatosis with polyangiitis; pSS: primary Sjögren syndrome; PR3: proteinase 3; ANCA: antineutrophil cytoplasmic antibody; C: cytoplasmic; MPO: myeloperoxidase; P: perinuclear.

**Table 4:** Application of the ACR/EULAR criteria for EGPA to patients with pSS presenting signs and symptoms suggestive of small- and medium-vessel vasculitis (*n* = 116).

Variables		Values
At pSS diagnosis	**Score**	**(*n* (%))**
Items for the ACR/EULAR criteria for EGPA and assigned scores to each item		
Clinical criteria		
Obstructive airway disease	+3	15 (12.9)
Nasal polyps	+3	1 (0.9)
Mononeuritis multiplex	+1	5 (4.3)
Laboratory, imaging, and biopsy criteria		
Serum eosinophil count ≥ 1000/μL	+5	8 (6.9)
Extravascular eosinophilic-predominant inflammation on biopsy	+2	0 (0.0)
PR3-ANCA (or C-ANCA) positivity	−3	4 (3.4)
Hematuria	−1	78 (67.2)
Patients with total score ≥ 6 (*n* (%))		2 (1.7)

ACR: American College of Rheumatology; EULAR: European Alliance of Associations for Rheumatology; EGPA: eosinophilic granulomatosis with polyangiitis; pSS: primary Sjögren syndrome; PR3: proteinase 3; ANCA: antineutrophil cytoplasmic antibody; C: cytoplasmic.

**Table 5:** Comparison of variables at pSS diagnosis between patients with OvSD/pSS/AAV and those without.

Variables	Patients with OvSD/pSS/AAV (*n* = 35)	Patients Without OvSD/pSS/AAV (*n* = 81)	*p*-Value
At pSS diagnosis			
Signs and symptoms suggestive of small- or medium-vessel vasculitis (*n*, (%))			
Lung parenchymal lesions supporting AAV	28 (80.0)	67 (82.7)	0.73
Pulmonary fibrosis or ILD on chest imaging	27 (77.1)	62 (76.5)	0.94
Pulmonary nodules, mass, and cavitation on chest imaging	23 (65.7)	42 (51.9)	0.17
Peripheral neuropathy	6 (17.1)	6 (7.4)	0.11
Suspected renal vasculitis	13 (37.1)	24 (29.6)	0.43
Demographic data			
Age (years)	57.0 (48.0–64.0)	55.0 (50.5–61.5)	0.42
Female gender (*n*, (%))	29 (82.9)	72 (88.9)	0.37
Items of the 2016 ACR/EULAR criteria for pSS (*n*, (%))			
Labial salivary gland with focal lymphocytic sialadenitis and focus score of ≥1 foci/4 mm^2^	2 (5.7)	3 (3.7)	0.63
Anti-SSA/Ro positive	35 (100.0)	76 (93.8)	0.13
Ocular staining score ≥ 5 in at least 1 eye	1 (2.9)	0 (0.0)	0.13
Schirmer’s test ≤ 5 mm/5 min in at least 1 eye	23 (65.7)	49 (60.5)	0.60
Unstimulated whole saliva flow rate < 0.1 mL/minute	23 (65.7)	60 (74.1)	0.36
ANCA positive (*n*, (%))			
ANCA positive	29 (82.9)	1 (1.2)	<0.001
MPO-ANCA (or P-ANCA) positive	27 (77.1)	1 (1.2)	<0.001
PR3-ANCA (or C-ANCA) positive	4 (11.4)	0 (0.0)	0.002
Laboratory results			
White blood cell count (/mm^3^)	5330.0 (4540.0–7000.0)	6260.0 (4825.0–8310.0)	0.093
Hemoglobin (g/dL)	12.3 (11.4–13.7)	12.8 (11.7–14.1)	0.39
Platelet count (×1000/mm^3^)	235.0 (169.0–293.0)	256.0 (199.5–321.0)	0.12
Blood urea nitrogen (mg/dL)	14.2 (10.8–18.7)	14.0 (10.1–16.9)	0.30
Serum creatinine (mg/dL)	0.7 (0.7–0.9)	0.7 (0.6–0.8)	0.11
Aspartate aminotransferase (IU/L)	25.0 (20.0–33.0)	24.0 (20.5–34.5)	0.63
Alanine aminotransferase (IU/L)	19.0 (14.0–31.0)	19.0 (14.0–33.0)	0.63
Total protein (g/dL)	7.4 (7.0–8.2)	7.4 (6.9–7.8)	0.29
Serum albumin (g/dL)	4.1 (3.6–4.4)	4.1 (3.8–4.4)	0.89
Acute-phase reactants			
ESR (mm/h)	44.0 (25.0–79.0)	48.0 (27.0–75.0)	0.67
CRP (mg/L)	2.9 (0.5–6.7)	1.7 (0.8–4.4)	0.52
Complement protein			
C3 (mg/dL)	104.4 (91.0–122.3)	106.5 (97.6–117.1)	0.54
C4 (mg/dL)	23.3 (13.2–28.1)	24.1 (18.2–29.8)	0.37

Values are expressed as a median (Q1–Q3) or *n* (%). pSS: primary Sjögren syndrome; OS: overlap syndrome; AAV: ANCA-associated vasculitis; ANCA: antineutrophil cytoplasmic antibody; ILD: interstitial lung disease; ACR: American College of Rheumatology; EULAR: European Alliance of Associations for Rheumatology; MPO: myeloperoxidase; P: perinuclear; PR3: proteinase 3; C: cytoplasmic; ESR: erythrocyte sedimentation rate; CRP: C-reactive protein; C3: complement 3; C4: complement 4.

## Data Availability

The dataset collected and/or analyzed in the present study is available on request from the corresponding author. This study expands upon preliminary findings presented at the EULAR 2024 Congress [34].

## Supplementary Materials

The following supporting information can be downloaded at https://www.mdpi.com/article/10.3390/diagnostics15091099/s1: Table S1: Itemized analysis of pSS patients reclassified as having OvSD/pSS/MPA and OvSD/pSS/GPA simultaneously according to the ACR/EULAR criteria for MPA, GPA, or EGPA; Table S2: Itemized analysis of pSS patients reclassified as having OvSD/pSS/MPA and OvSD/pSS/EGPA simultaneously according to the ACR/EULAR criteria for MPA, GPA, or EGPA; Table S3: Itemized analysis of pSS patients who did not have ANCA but were reclassified as having OvSD/pSS/GPA according to the ACR/EULAR criteria for GPA; Table S4: Itemized analysis of pSS patients who did not have ANCA but were reclassified as having OvSD/pSS/EGPA according to the ACR/EULAR criteria for EGPA; Table S5: Itemized analysis of pSS patients who had ANCA but were not reclassified as having OvSD/pSS/MPA, OvSD/pSS/GPA, or OvSD/pSS/EGPA according to the ACR/EULAR criteria for MPA, GPA, or EGPA.

## Abbreviations

The following abbreviations are used in this manuscript:

AAV	antineutrophil cytoplasmic antibody-associated vasculitis
ACR	American College of Rheumatology
ANCA	antineutrophil cytoplasmic antibody
C	cytoplasmic
C3	complement 3
C4	complement 4
CRP	C-reactive protein
EGPA	eosinophilic granulomatosis with polyangiitis
ESR	erythrocyte sedimentation rate
EULAR	European Alliance of Associations for Rheumatology
GN	glomerulonephritis
GPA	granulomatosis with polyangiitis
ILD	interstitial lung disease
MPA	microscopic polyangiitis
MPO	myeloperoxidase
OS	overlap syndrome
P	perinuclear
PR3	proteinase 3
pSS	primary Sjögren syndrome
TIN	tubulointerstitial nephritis
