# Supplementary material for: Overlap Syndrome of Primary Sjögren Syndrome with Antineutrophil Cytoplasmic Antibody (ANCA)-Associated Vasculitis Based on the American College of Rheumatology (ACR)/European Alliance of Associations for Rheumatology (EULAR) Criteria
Source: Diagnostics (Basel). 2025 Apr 25;15(9):1099. doi: 10.3390/diagnostics15091099 (PMC12071592; doi:10.3390/diagnostics15091099)
Supplement: Supplementary file 1 [file diagnostics-15-01099-s001.zip › SUPPLEMENTARY TABLE S1(OS-pSS-AAV).pdf]

Supplementary Table S1. Itemized analysis of pSS patients reclassified as having OvSD/pSS/MPA and OvSD/pSS/GPA simultaneously according to the ACR/EULAR criteria for MPA, GPA, or EGPA

Patient's number	Scores based on the 2022 ACR/EULAR criteria for MPA	1 (-3)	2 (+6)	3 (+3)	4 (+3)	5 (-1)	6 (-4)
8	6	1	1	0	1	0	0
9	9	0	1	1	0	0	0
10	8	0	1	1	0	1	0
11	8	0	1	1	0	1	0

1 = Nasal involvement (discharge, ulcers, crusting, congestion, septal defect/perforation); 2 = MPO-ANCA (or P-ANCA) positivity; 3 = Fibrosis or interstitial lung disease on chest imaging; 4 = Pauci-immune glomerulonephritis on biopsy; 5 = PR3-ANCA (or C-ANCA) positivity; 6 = Serum eosinophil count ≥ 1000/μL

Patient's number	Scores based on the 2022 ACR/EULAR criteria for GPA	1 (+3)	2 (+2)	3 (+1)	4 (+5)	5 (+2)	6 (+2)	7 (+1)	8 (+1)	9 (-1)	10 (-4)
8	6	1	1	0	0	0	0	1	1	1	0
9	5	0	1	0	0	1	1	0	0	1	0
10	7	0	0	0	1	1	0	1	0	1	0
11	8	0	1	0	1	1	0	0	0	1	0

1 = Nasal involvement (discharge, ulcers, crusting, congestion, septal defect/perforation); 2 = Cartilaginous involvement; 3 = Conductive or sensorineural hearing loss; 4 = PR3-ANCA (or C-ANCA) positivity; 5 = Pulmonary nodules, mass, or cavitation; 6 = Granuloma, granulomatous inflammation, or giant cells on biopsy; 7 = Nasal/paranasal sinusitis or mastoiditis on imaging; 8 = Pauci-immune glomerulonephritis on biopsy; 9 = MPO-ANCA (or P-ANCA) positivity; 10 = Serum eosinophil count ≥ 1000/μL

Patient's number	Scores based on the 2022 ACR/EULAR criteria for EGPA	1 (+3)	2 (+3)	3 (+1)	4 (+5)	5 (+2)	6 (-3)	7 (-1)
8	-1	0	0	0	0	0	0	1
9	0	0	0	0	0	0	0	0
10	0	1	0	1	0	0	1	1
11	-4	0	0	0	0	0	1	1

1 = obstructive airway disease; 2 = nasal polyps; 3 = mononeuritis multiplex; 4 = Serum eosinophil count ≥ 1000/μL; 5 = Extravascular eosinophilic predominant inflammation on biopsy; 6 = PR3-ANCA (or C-ANCA) positivity; 7 = haematuria

pSS: primary Sjögren syndrome; OS: overlap syndrome; MPA: microscopic polyangiitis; GPA: granulomatosis with polyangiitis; ACR: the American College of Rheumatology; EULAR: the European Alliance of Associations for Rheumatology; EGPA: eosinophilic GPA; MPO: myeloperoxidase; ANCA: antineutrophil cytoplasmic antibody; P: perinuclear; PR3: proteinase 3; C: cytoplasmic.
