# Supplementary material for: Overlap Syndrome of Primary Sjögren Syndrome with Antineutrophil Cytoplasmic Antibody (ANCA)-Associated Vasculitis Based on the American College of Rheumatology (ACR)/European Alliance of Associations for Rheumatology (EULAR) Criteria
Source: Diagnostics (Basel). 2025 Apr 25;15(9):1099. doi: 10.3390/diagnostics15091099 (PMC12071592; doi:10.3390/diagnostics15091099)
Supplement: Supplementary file 1 [file diagnostics-15-01099-s001.zip › SUPPLEMENTARY TABLE S3(OS-pSS-AAV).pdf]

Supplementary Table S3. Itemized analysis of pSS patients who did not have ANCA but were reclassified as having OvSD/pSS/GPA according to the ACR/EULAR criteria for GPA

**Patient's number**	**Scores based on the 2022 ACR/EULAR criteria for GPA**	**1 (+3)**	**2 (+2)**	**3 (+1)**	**4 (+5)**	**5 (+2)**	**6 (+2)**	**7 (+1)**	**8 (+1)**	**9 (-1)**	**10 (-4)**
2	7	1	0	0	0	1	1	0	0	0	0
3	5	0	1	0	0	1	0	1	0	0	0
4	7	1	1	0	0	1	0	0	0	0	0
5	6	1	0	0	0	1	0	1	0	0	0
6	6	1	0	0	0	1	0	1	0	0	0

1 = Nasal involvement (discharge, ulcers, crusting, congestion, septal defect/perforation); 2 = Cartilaginous involvement; 3 = Conductive or sensorineural hearing loss; 4 = PR3-ANCA (or C-ANCA) positivity; 5 = Pulmonary nodules, mass, or cavitation; 6 = Granuloma, granulomatous inflammation, or giant cells on biopsy; 7 = Nasal/paranasal sinusitis or mastoiditis on imaging; 8 = Pauci-immune glomerulonephritis on biopsy; 9 = MPO-ANCA (or P-ANCA) positivity; 10 = Serum eosinophil count ≥ 1000/μL

pSS: primary Sjögren syndrome; ANCA: antineutrophil cytoplasmic antibody; OS: overlap syndrome; GPA: granulomatosis with polyangiitis; ACR: the American College of Rheumatology; EULAR: the European Alliance of Associations for Rheumatology; PR3: proteinase 3; C: cytoplasmic; MPO: myeloperoxidase; P: perinuclear.
