# Supplementary material for: Overlap Syndrome of Primary Sjögren Syndrome with Antineutrophil Cytoplasmic Antibody (ANCA)-Associated Vasculitis Based on the American College of Rheumatology (ACR)/European Alliance of Associations for Rheumatology (EULAR) Criteria
Source: Diagnostics (Basel). 2025 Apr 25;15(9):1099. doi: 10.3390/diagnostics15091099 (PMC12071592; doi:10.3390/diagnostics15091099)
Supplement: Supplementary file 1 [file diagnostics-15-01099-s001.zip › SUPPLEMENTARY TABLE S4(OS-pSS-AAV).pdf]

Supplementary Table S4. Itemized analysis of pSS patients who did not have ANCA but reclassified as having OvSD/pSS/EGPA according to the ACR/EULAR criteria for EGPA

Patient's number	Scores based on the 2022 ACR/EULAR criteria for EGPA	1 (+3)	2 (+3)	3 (+1)	4 (+5)	5 (+2)	6 (-3)	7 (-1)
7	8	1	0	0	1	0	0	0

1 = obstructive airway disease; 2 = nasal polyps; 3 = mononeuritis multiplex; 4 = Serum eosinophil count ≥ 1000/μL; 5 = Extravascular eosinophilic predominant inflammation on biopsy; 6 = PR3-ANCA (or C-ANCA) positivity; 7 = haematuria

pSS: primary Sjögren syndrome; ANCA: antineutrophil cytoplasmic antibody; OS: overlap syndrome; EGPA: eosinophilic granulomatosis with polyangiitis; ACR: the American College of Rheumatology; EULAR: the European Alliance of Associations for Rheumatology; PR3: proteinase 3; C: cytoplasmic.
